# Enhanced heat transfer is dependent on thickness of graphene films: the heat dissipation during boiling

**DOI:** 10.1038/srep06276

**Published:** 2014-09-03

**Authors:** Ho Seon Ahn, Jin Man Kim, TaeJoo Kim, Su Cheong Park, Ji Min Kim, Youngjae Park, Dong In Yu, Kyoung Won Hwang, HangJin Jo, Hyun Sun Park, Hyungdae Kim, Moo Hwan Kim

**Affiliations:** 1 Division of Mechanical System Engineering, Incheon National University, Incheon, Republic of Korea; 2 Division of Advanced Nuclear Engineering, POSTECH, Pohang, Republic of Korea; 3 Korea Atomic Energy Research Institute, Daejeon, Republic of Korea; 4 Department of Mechanical Engineering, POSTECH, Pohang, Republic of Korea; 5 Department of Nuclear Engineering, Kyung Hee University, Yongin, Republic of Korea; 6 These authors contributed equally to this work.

## Abstract

Boiling heat transfer (BHT) is a particularly efficient heat transport method because of the latent heat associated with the process. However, the efficiency of BHT decreases significantly with increasing wall temperature when the critical heat flux (CHF) is reached. Graphene has received much recent research attention for applications in thermal engineering due to its large thermal conductivity. In this study, graphene films of various thicknesses were deposited on a heated surface, and enhancements of BHT and CHF were investigated via pool-boiling experiments. In contrast to the well-known surface effects, including improved wettability and liquid spreading due to micron- and nanometer-scale structures, nanometer-scale folded edges of graphene films provided a clue of BHT improvement and only the thermal conductivity of the graphene layer could explain the dependence of the CHF on the thickness. The large thermal conductivity of the graphene films inhibited the formation of hot spots, thereby increasing the CHF. Finally, the provided empirical model could be suitable for prediction of CHF.

Following the demonstration by Geim and Novoselov^1^ that graphene forms a two-dimensional honeycomb-monolayer, graphene has attracted considerable attention from the research community because of its large thermal conductivity^2^,^3^,^4^, high transparency^5^, very large charge carrier mobility^6^, and favorable mechanical properties^7^. Facile and simple synthesis routes for graphene that are suitable for mass-production have been identified for applications in mechanical, chemical, and biomimetic fields^8^,^9^. Aqueous graphene has received much attention for a range of potential applications, including nanoelectronics, sensors, batteries, and supercapacitors^10^. Aqueous suspensions of graphene have been synthesized via the oxidation and subsequent reduction of graphene. Using a chemical process involving hydrazine, graphene oxide (GO) can be transformed into reduced graphene oxide (RGO), which exhibits similar properties as graphene. For example, aqueous graphene may be formed into a thin film via filtration^10^,^11^.

Since graphene typically exists in the form of ultra-thin microscale flakes, much effort has been invested to synthesize large-area films that retain the properties of graphene. Jang *et al.*^12^ found that the thermal conductivity of graphene film on a silicon substrate increased as the film became thicker: as the thickness increased from 1 to 100 nm, the thermal conductivity increased from 110 to 1100 W K^−1^m^−1^ (at room temperature). Although the thermal conductivity of these graphene films is significantly smaller than has been reported for single graphene flakes, it remains larger than that of many other substrates, and hence graphene remains attractive for heat transfer applications. However, despite the favorable heat transport properties, little information about applications of graphene on thermal system is available. The characteristics of a heated surface, including the surface wettability^13^,^14^,^15^, capillary wicking^16^,^17^, the surface factor^18^,^19^, and the enhanced surface area (fin-effect)^20^, can strongly influence boiling heat transfer (BHT) and the critical heat flux (CHF). The latter is the limiting factor for BHT. Several literatures in past reported that the thermal properties of the heater surface might affect strongly both the BHT and CHF^21^,^22^, i.e., that the thermal conductivity and capacity of the heater surface influence BHT and the CHF.

Here, we report that thin graphene films on a heated surface may result in more efficient dissipation of heat in the lateral direction, which inhibits the formation of dry/hot spots during boiling, leading to an increase and CHF. The role of graphene films on the heated surface was investigated using infrared high-speed visualization of the boiling process. We found that the graphene layer led to an increase in the CHF via lateral heat transport, which inhibited the formation of hot/dry spots. Pool-boiling experiments were carried out in the Pohang University of Science and Technology (POSTECH) pool-boiling experimental facility, which is illustrated in the schematic diagram shown in Fig. S1. A silicon substrate with a deposited Pt film was used as the heated surface (see Figs. S2 and S3). The RGO colloid (see Fig. S4) of 0.001 wt. % was prepared via the modified Hummers' method^10^, which is a well-known synthesis route used to form water-soluble RGO flakes. The RGO flakes were characterized using transmission electron microscopy, TEM (JEM-2200FS, 0.1 nm-200 KeV, JEOL), selected-area electron diffraction, SAED (JEM-2200FS, 0.1 nm-200 KeV, JEOL), and atomic force microscopy, AFM (Dimension 3100, 0.1 nm(X-Y), 0.01 nm(Z), VEECO). The RGO colloidal dispersion appeared black in color (see Fig. S4a). The AFM image shown in Fig. S4d reveals that the flakes were irregularly shaped, approximately 1–2 μm across and 1–20 nm thick.

The RGO colloidal suspension was filtered to prepare the graphene films (see Fig. S5), the thickness of which was predicted based on the following relationship for relative thickness regulation: 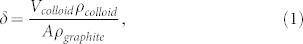
where *V_colloid_* is the volume of the RGO flakes, *A* is the area of the film, *δ* is the thickness of the film, *ρ_graphite_* is the density of the graphite, and *ρ_colloid_* is the density of the RGO colloid. The filtered graphene film was transferred to the surface of the silicon heater (see Fig. S2) and attached using compression bonding. Following drying in a vacuum oven at 63°C for 1 hour, the filter paper was removed from the heater surface (see Fig. S6). The thickness of the graphene film was characterized using AFM (Fig. S7), and found to be the range 40–200 nm. After graphene film was transferred on the surface of the silicon heater, the surface was characterized using scanning electron microscopy, SEM (JSM-7401F, 1.0 nm-5 kV, JEOL). Figure 1a shows SEM images of the transferred film, which reveal that the RGO flakes were stacked and horizontally aligned.

Figure 1b shows the boiling curves of the heated surface with and without the transferred graphene layer. The onset of nucleate boiling (ONB) occurred at a similar point for all the surfaces, and the graphene-coated surfaces exhibited an increase in both BHT and the CHF compared with the bare silicon surface. BHT was more significant on the graphene films than on the bare silicon surface, and accounted for as much as 90%. The BHT component was similar for the 50-, 100-, 150- and 200-nm-thick films; however, the respective CHFs of the four different thickness films were 1102, 1204, 1302 and 1300 kW m^−2^. The CHF increased with the thickness of the graphene film.

Improved surface wettability^13^,^14^,^15^, increased surface area, and capillary wicking due to micro/nano structures^16^,^17^ may result in an increase in the CHF. However, the results cannot be explained from above reasons. Figure 1c shows static contact angle measurements of the surfaces. The contact angle can be different depending on its circumstance, so the contact angles were measured using Smart Drop which is a contact angle measurement device with the same condition of temperature and humidity. The bare silicon surface exhibited a contact angle of 63°, and the surfaces with 100- and 200-nm-thick graphene films exhibited contact angles of 81.9° and 81.5°, respectively. The static contact angle of the graphene surface was larger than the bare silicon surface. Because graphene is hydrophobic^23^, we may expect that the film RGO flakes with carboxyl groups will also be hydrophobic^11^. The change in the surface wettability due to the presence of the graphene film cannot explain the increase in the CHF, as there was no significant change in the contact angle as the thickness of the graphene film was varied. However, the slight decrease in wettability compared with the bare silicon surface may enhance BHT because the diameter of departing bubbles during nucleate boiling is expected to decrease.

The local temperature profile of the bare silicon and 50-nm-thick graphene-coated surfaces at 40% of the CHF was investigated using infrared (IR) high-speed visualization (see section S5 and Fig. S12 in the supplementary information for details of the IR visualization technique). The graphene-coated surface exhibited a larger nucleation site density and bubble frequency, with a smaller bubble diameter. These differences result from the surface characteristics of the graphene film, and lead to enhanced boiling performance, as shown in Fig. 1b. The heat flux due to boiling can be described as follows^24^: 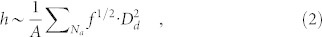
where *f* is the frequency of bubble departure, *D_d_* is the bubble diameter, and *N_a_* is the density of nucleation sites. As shown in Fig. 1a, the graphene films exhibited nanometer-scale folded edges, where the edges of the upper-most RGO flakes were partially folded; this is consistent with the observations of Bae^25^. It follows that bubble nucleation on the graphene film may occur more readily than on the bare silicon heater because the nanometer-scale folded edges of the RGO flakes may act as nucleation sites. Thus, the density of nucleation sites was larger, and the diameter of the departing bubbles was smaller on the graphene surface. Based on Eq. 2, we can expect a factor of 1.8 increase in the boiling heat transfer rate on the graphene-coated surface compared with the bare silicon surface.

It is well known that an increase in the thermal conductivity of the heated surface can lead to an increase in the CHF. This is due to lateral heat dissipation, which can inhibit the formation of hot spots during nucleate boiling^22^. It has been reported that the density *ρ_w_*, as well as the thermal conductivity *k_w_*, the heat capacity *C_p,w_*, and the thickness of the heated surface *δ_w_* can all affect the CHF^18^,^19^. Arik and Bar-Cohen^24^ reported that the CHF increases with the thickness of the heated surface, approaching an asymptotic limit. They found that the thermal activity could be described by 
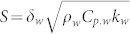
, and that the ratio of the CHF q″*_CHF_* to the asymptote q″*_max_* can described by 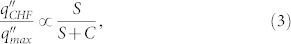
where the constant *C* is a fitting parameter. We used a phenomenological relationship between the thermal conductivity of the graphene film and the thickness^12^ to calculate the thermal activity. Because the thicknesses of the graphene films were in the range 15–200 nm, the effective thermal conductivity of the graphene films was estimated to be in the range 1000–2000 W·m^−1^·K^−1^ (see Fig. S8). The thermal capacity and density of graphene were assumed to be the same as those of graphite^26^. As the thickness of the graphene films increased to 150 nm, the CHF increased to 1320 kW·m^−2^. When the thickness of graphene increased beyond 150 nm, no further increase in the CHF was observed (see Fig. S9). This asymptotic behavior of the CHF as a function of the thickness of the graphene film indicates that the maximum CHF was 1320 kW·m^−2^, which represents a 62.5% increase compared with the bare silicon surface. As shown in Fig. S8, the thermal conductivity of graphene film is already close to asymptotic value at the 40-nm-thick garphene film. Thus, the increase in the CHF as the thickness varied from 40–200 nm is dependent only on the thickness of the graphene films. We calculated the thermal activity of the graphene films, which were in the range 0 < *S* < 1; however, most were of the order of 10^−2^. The fitting constant was varied in the range 0.0001 < *C* < 0.001, as shown in Fig. 2a, which was found to provide good agreement with the experimental data. In investigations of the CHF using thin-films of other materials^19^,^27^, the CHF approached an asymptotic limit as the thickness increased. In this study, we found that this asymptote occurred when the thickness of the graphene films was 150 nm. For comparison, with copper thin films^27^, this asymptotic limit was found to occur when the thickness of the layer was 25 μm.

The bare silicon and graphene-coated surfaces were visualized during boiling at 98% of the CHF using a high-speed IR camera (Fig. 2b). A large dry area (shown by the bright region in the figure), which persisted for more than 100 ms, was observed on the bare silicon surface. This dry area appeared as a hot circular area, which expanded as the microlayer evaporated at the center. As the CHF is reached, such dry areas become too hot to be re-wetted^28^,^29^,^30^. The graphene-coated surface exhibited only nucleate boiling at the same heat flux. No large dry areas were observed on the graphene-coated surface at the same heat flux. The graphene film has a larger thermal activity, and so can more effectively dissipate heat in the lateral direction, so the CHF was larger on the graphene-coated surface. Lateral heat dissipation in the graphene film is related not only to the thermal conductivity of the graphene film, but also to the thickness in Fig. 2a. An increase in the thermal activity may inhibit the formation of hot/dry areas. Figures 3a and 3b show IR images that compare graphene-coated heated surfaces of different thicknesses (and therefore different thermal activities). Large dry areas were less likely to appear and were shorter lived on the 50-nm-thick graphene-coated heated surface than on the 15-nm-thick graphene-coated surface. The 50-nm-thick graphene film can dissipate heat at a greater rate than the 15-nm-thick graphene film, since the thermal activity is related to the thickness. Thus, lateral heat dissipation in the graphene film enhanced CHF.

To gain further insight into lateral heat dissipation in the graphene films, the surface temperature profile of the hot/dry areas was investigated using IR visualization at 98% of the CHF, as shown in Figs. 4a and 4b, as well as Fig. S13 in the supplementary information. The temperature profiles are shown during the bubble growth period, just before the interface (base line) of the bubbles became distorted, i.e., when the triple line of the bubbles is expected to induce diffraction of the infrared light as a result of a thicker microlayer during bubble growth due to buoyancy but not departure^31^. The bare silicon heated surface exhibited a steep spatial temperature gradient; however, the graphene-coated surfaces exhibited smaller spatial temperature gradients. The thermal activity increased with the thickness of the graphene layer, which is directly related to the lateral heat dissipation, as shown by the spatial temperature gradients in Fig. 4b.

The thermal diffusivity is given by 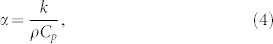
where *k* is the thermal conductivity, *ρ* is the density, and *C_p_* is the heat capacity. In a material with a large thermal diffusivity, heat moves rapidly because the substance conducts heat quickly relative to its volumetric heat capacity. Based on the IR data describing the spatial temperature gradient and the changes in temperature over time (see Fig. 4c), we can estimate the thermal diffusivity using the following relationship^32^: 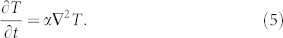
In this step, we extracted one-dimensional temperature profiles with assumption which temperature distribution was same in radial direction because these bubble sites were well defined circularly. We found that the thermal diffusivity of the bare silicon heated surface was *α_exp_bare_* = 7.448 × 10^−5^ m^2^·s^−1^, and that of the 50-nm-thick graphene-carted surface was *α_exp__*_50*nm*_ = 9.021 × 10^−5^ m^2^·s^−1^. This value of the thermal diffusivity of silicon is in good agreement with the previously reported value of 7.974 × 10^−5^ m^2^·s. The data used to calculate these thermal diffusivities is listed in Tables S1 and S2. The increased thermal diffusivity allows heat to be transported more rapidly, leading to the suppression of local hot/dry spots under the bubbles.

In summary, the graphene-coated heater showed an increase in BHT and CHF. As the thickness of the graphene films increased, the CHF also increased up to an asymptotic limit when the graphene layer was approximately 150 nm thick. The increased BHT was explained by the slight decrease in the wettability and the folded edges of the RGO flakes, which led to a decrease in the diameter of the departing bubbles, a larger bubble generation frequency, and an increase in the density of the bubble nucleation sites. The increase in the CHF was explained by considering the thermal activity of the graphene films, and the dependence thereof on the thickness and thermal properties of the layer, as well as the thermal diffusivity, which was calculated based on high-speed IR visualization data.

## Methods

### infrared high-speed visualization

The local temperature distribution of the surface was acquired using the IR thermometry technique illustrated in Fig. S10. A 700-nm-thick IR-opaque indium–tin–oxide (ITO) layer was deposited on the silicon heater. A high-speed IR camera (FLIR SC6000) was placed below the heater, and the working fluid was located on top of the heater. When boiling occurred, the IR camera measured the temperature distribution of the thin ITO layer though the IR-transparent silicon heater. The spatial resolution of the system was 65 µm, and the temporal resolution was 1 ms.

### boiling experimental procedure

Prior to each experiment, deionized (DI) water was boiled for 1 hour using a cartridge heater to degas the water. Two wires were soldered to each electrode to apply a voltage and measure the temperature using the correlation shown in Fig. S3a. The heat flux data were recorded using an Agilent 34970A data acquisition system, and the surface temperatures were calculated at the same time. After the water had boiled for more than one hour, the heat flux was increased in steps of 100 kW·m^−2^, maintaining saturated conditions (100°C and 1 atm) for 2 minutes during each step of the experiment. At large heat fluxes, the power supply was shut down when a sudden increase in the wall temperature was observed, which corresponded to the CHF.

### DATA EXTRACTION From the infrared high-speed visualization

All images of the infrared high-speed visualization were trimmed and processed using Matlab and Image J. Raw image data appeared too dark, and so nucleation could not be observed. Therefore, the brightness and contrast were adjusted for each experimental case; this adjustment did not result in a change in the recorded temperature. One- and two-dimensional data arrays of the temperature distribution were extracted for the selected domain to analyze the temperature distribution and the changes with time thereof. The overall temperature distribution, one-dimensional temperature profiles, and temperature plots were obtained from the extracted data.

## Author Contributions

H.S.A. designed the experiments and all procedures, and was the main author of the paper. J.M.K. carried out the experiments, provided the figure artwork, performed the data analysis, and contributed some written material to the manuscript. T.J. K. assisted with the analysis of the graphene film. S.C.P., J.M.K. and D.I.Y. also carried out experiments, fabricated experimental apparatus, and acquired and interpreted the SEM and TEM data. Y.P. carried out the visualization experiments using the infrared camera and fabricated some of the experimental apparatus. K.W.H. analyzed the AFM data. H.J.J. assisted with the analysis of the surface characteristics and boiling performance. H.S.P. assisted in the analysis of the IR visualization. H.K. and M.H.K. equally directed the project. All authors discussed the results and contributed to the paper.

## Supplementary Material

Supplementary Information Supplementary information

Supplementary Information Video clip of IR high-speed camera in real time

## Figures and Tables

**Figure 1:**
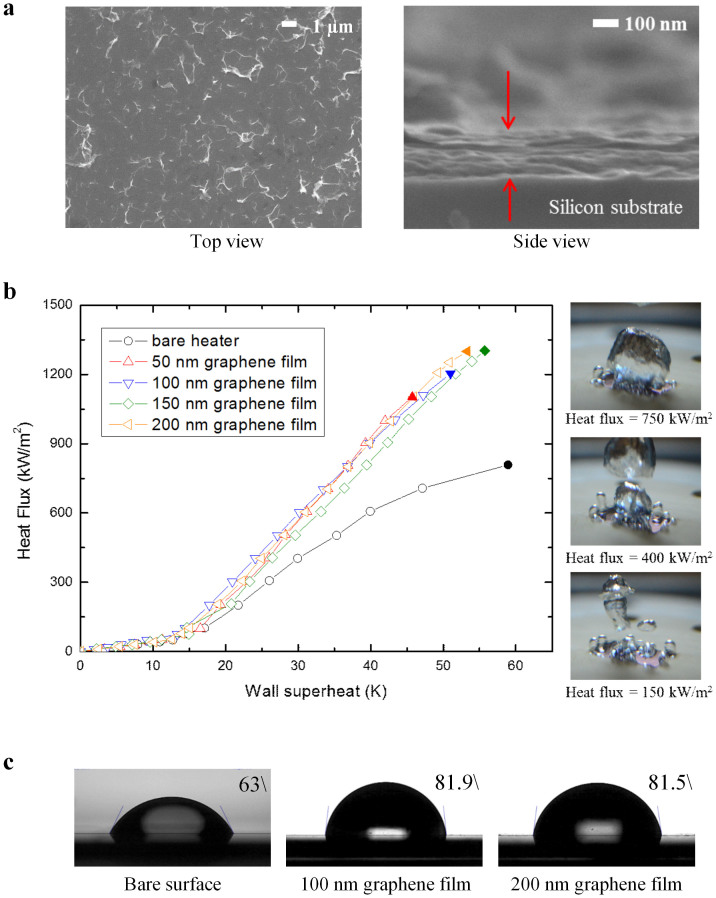
Heated surface analysis and boiling performance. (a), SEM images of the graphene film. (b), Boiling curves. (c), Static contact angle measurements.

**Figure 2:**
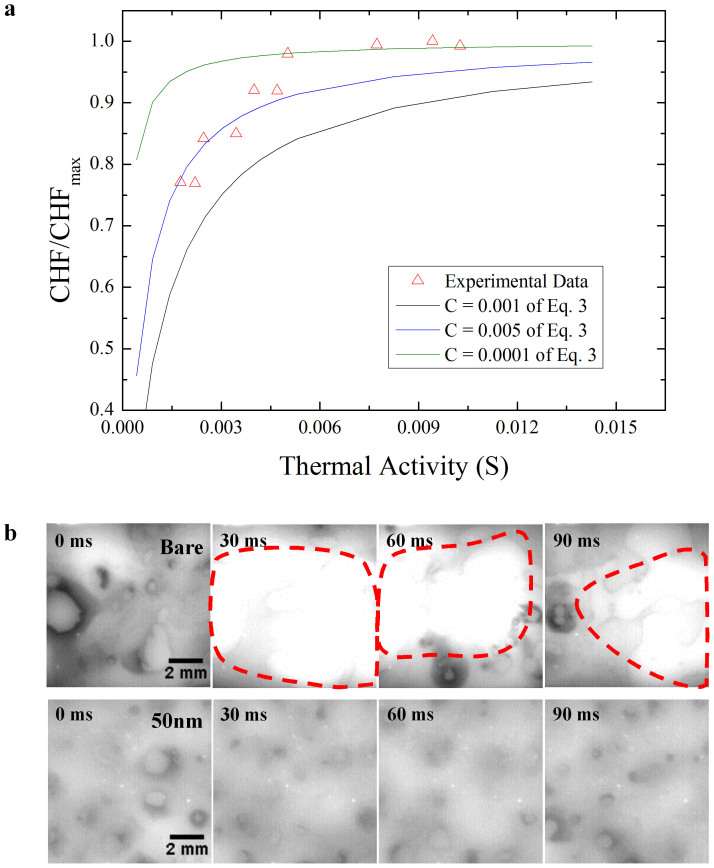
Thermal properties and the effects on boiling performance. (a), The critical heat flux as a function of the thermal activity. (b), IR visualizations of the bare silicon and the 50-nm-thick graphene-coated surfaces during boiling at 98% of the CHF of the bare silicon surface.

**Figure 3:**
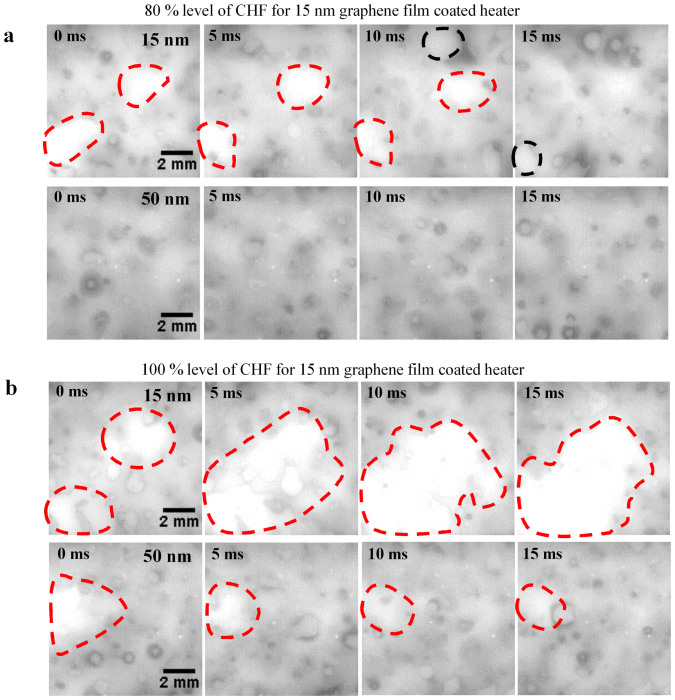
IR images during boiling. (a), The formation of dry areas on the 15- and 50-nm-thick graphene-coated surfaces at 80% of the CHF of the 15-nm-thick graphene-coated surface. (b), The formation of dry areas on the 15- and 50-nm-thick graphene-coated surfaces at the CHF of the 15-nm-thick graphene-coated surface.

**Figure 4:**
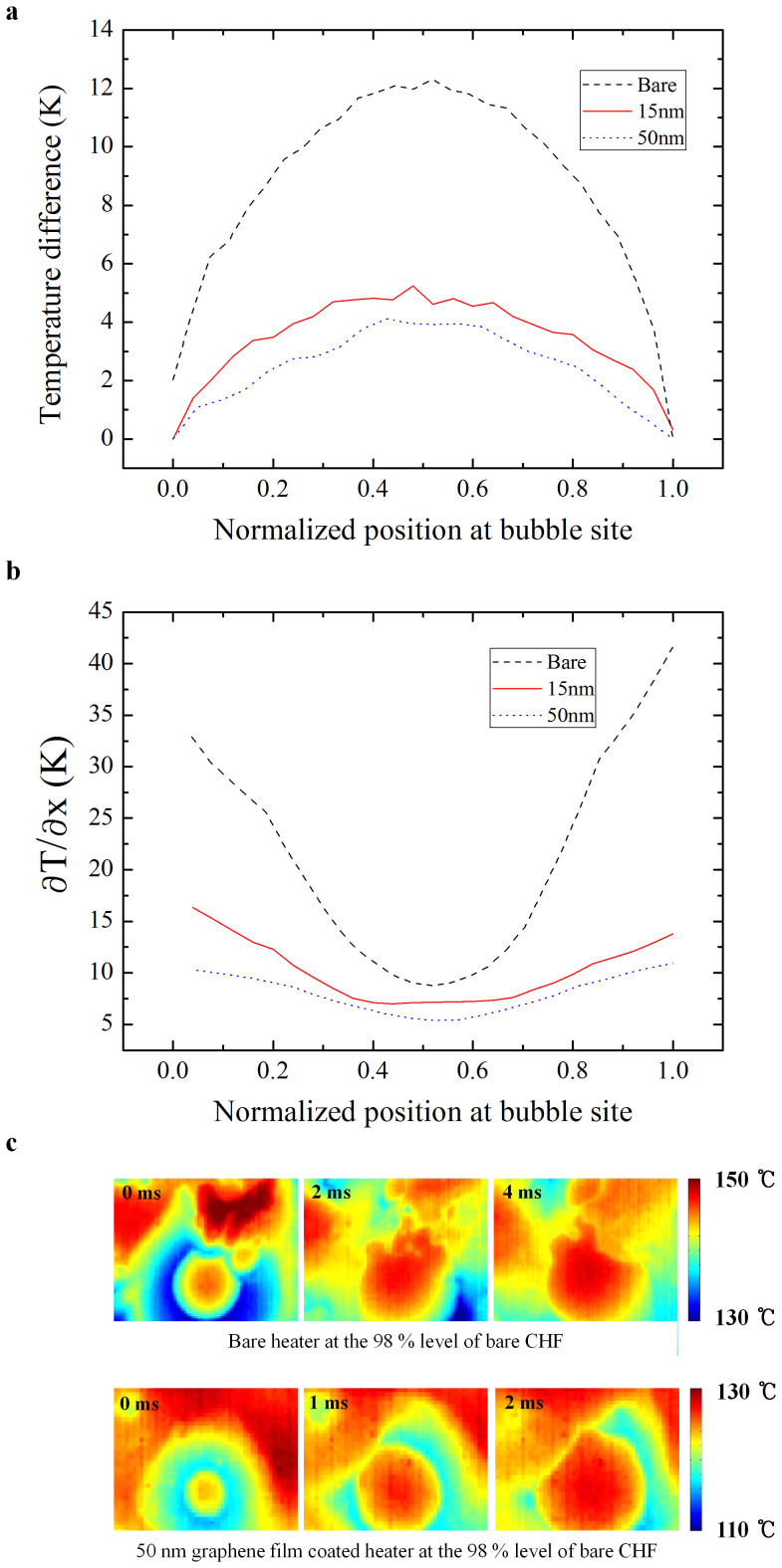
Surface temperature profiles. (a), Temperature profile of a bubble nucleation site at 98% of the CHF of the bare silicon surface. (b), Spatial gradient of the temperature of a bubble nucleation site at 98% of the CHF of the bare silicon surface. (c), Temperature profiles on the bare silicon surface and the 50-nm-thick graphene-coated surface at the same heat flux.
