# Supplementary material for: Enhanced heat transfer is dependent on thickness of graphene films: the heat dissipation during boiling
Source: Sci Rep. 2014 Sep 3;4:6276. doi: 10.1038/srep06276 (PMC4152752; doi:10.1038/srep06276)
Supplement: Supplementary Information [file srep06276-s1.pdf]

SUPPLEMENTARY INFORMATION

Enhanced heat transfer is dependent on thickness of graphene films: the heat dissipation during boiling

Ho Seon Ahn[1,*], Jin Man Kim[2,*], TaeJoo Kim[3], Su Cheong Park[4], Ji Min Kim[4], Youngjae Park[5], Dong In Yu[4], Kyoung Won Hwang[4], Hang Jin Jo[2], Hyun Sun Park[2], Hyungdae Kim[5] and Moo Hwan Kim[2]

[1]Division of Mechanical System Engineering, Incheon National University, Incheon, Republic of Korea

[2]Division of Advanced Nuclear Engineering, POSTECH, Pohang, Republic of Korea

[3]Korea Atomic Energy Research Institute, Daejeon, Republic of Korea

[4]Department of Mechanical Engineering, POSTECH, Pohang, Republic of Korea

[5]Department of Nuclear Engineering, Kyung Hee University, Yongin, Republic of Korea

[*]These authors equally contributed to this work.

Corresponding Authors: Professors, Hyungdae Kim (hdkims@khu.ac.kr) and Moo Hwan Kim (mhkim@postech.ac.kr)

S1. Pool-boiling experimental facility

Figure S1a shows a schematic diagram of the experimental pool-boiling facility, which was designed to carry out pool-boiling experiments to measure BHT and the CHF under atmospheric pressure and using Joule heating. The facility operates with a test sample and a main pool chamber. Here, the test sample consisted of a silicon substrate and a polyetheretherketone (PEEK) test sample frame. PEEK is a thermoplastic that has a large thermal resistance and is compatible with an aqueous chemical environment. The test samples were waterproofed and fixed using an adhesive sealant (Permatex clear room-temperature vulcanizing (RTV) silicone), as shown in Fig. S1b. The main pool chamber was a rectangular bath formed of 10-mm-thick aluminum sheet with a 4-liter capacity. The test sample was located at the bottom of the pool chamber. A reflux condenser was installed at the top of the main pool chamber to prevent evaporation of the working fluid, which was DI water.

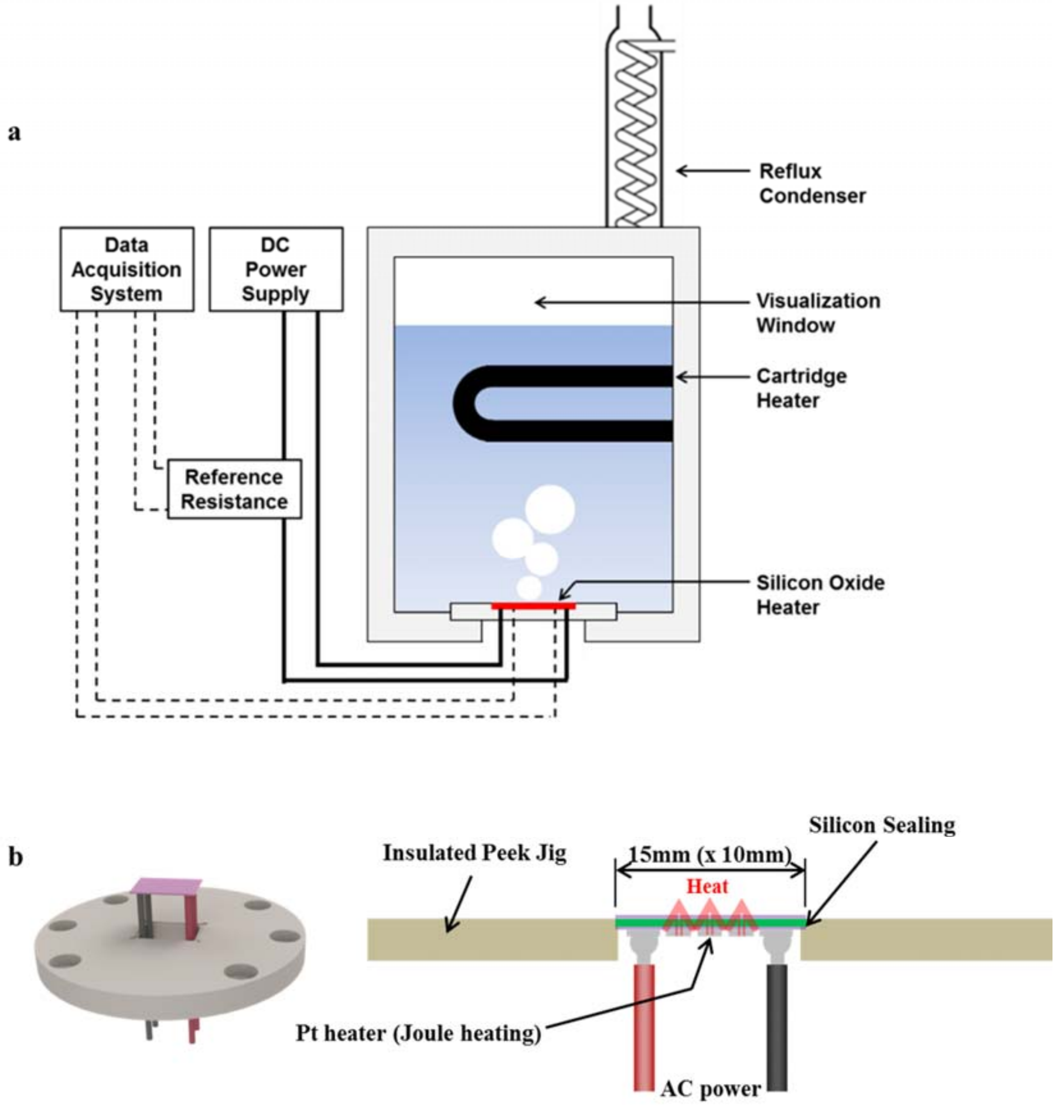

Figure S1 | Experimental facility. a, Pool boiling chamber. **b,** Test section.

S2. Heater

Figure S2 shows images of the heater used in this work, which consisted of a double-sided polished silicon substrate with a thermally grown silicon dioxide (SiO_2) surface and a deposited platinum thin-film heater. The test heater was composed of rectangular silicon wafer plate. The silicon substrate measured 25 × 20 mm, and had an SiO_2 layer to eliminate native oxidation effects on both the top and bottom surfaces. The heating element was a 120-nm-thick platinum thin film, which was deposited on the bottom surface using an electron-beam (E-beam) evaporator. A 12-nm-thick titanium thin film was used to form an adhesion layer between the silicon wafer and the platinum film. The platinum film heater was H-shaped (15 × 10 mm), and was the main heating area. Wire electrodes were attached using lead solder; the resistance of the electrodes was less than 1% of the total sample resistance. Joule heating was used to heat the sample. To calibrate the wall temperature, it is important to accurately determine the voltage and current during operation so that the resistance of each test sample can be correlated as a function of the temperature, as shown in Fig. S3a. A direct current (DC) power supply (120 V / 18 A) was used to power to the Joule heating system. The voltage applied to the test sample was measured directly using a power measuring line and a data acquisition system with a 1-second period. A reference resistance (Vishay, RH02510R00FC02) was immersed in a constant-temperature bath at 10°C, and used to calculate the current passing through the heater. The heat flux was calculated from the voltage applied to the heater V_{heater} and the voltage V_{ref} across the reference resistor, which had resistance R_{ref} , as follows

$$q'' = \frac{V_{heater} I_{circuit}}{A_{heater}} \quad , \qquad \text{S-1}$$

where $I_{circuit} = V_{ref} / R_{ref}$ is the current in the circuit and A_{heater} is the area of the heater. The resistance of the heater was found from $R_{heater} = V_{heater} / I_{circuit}$.

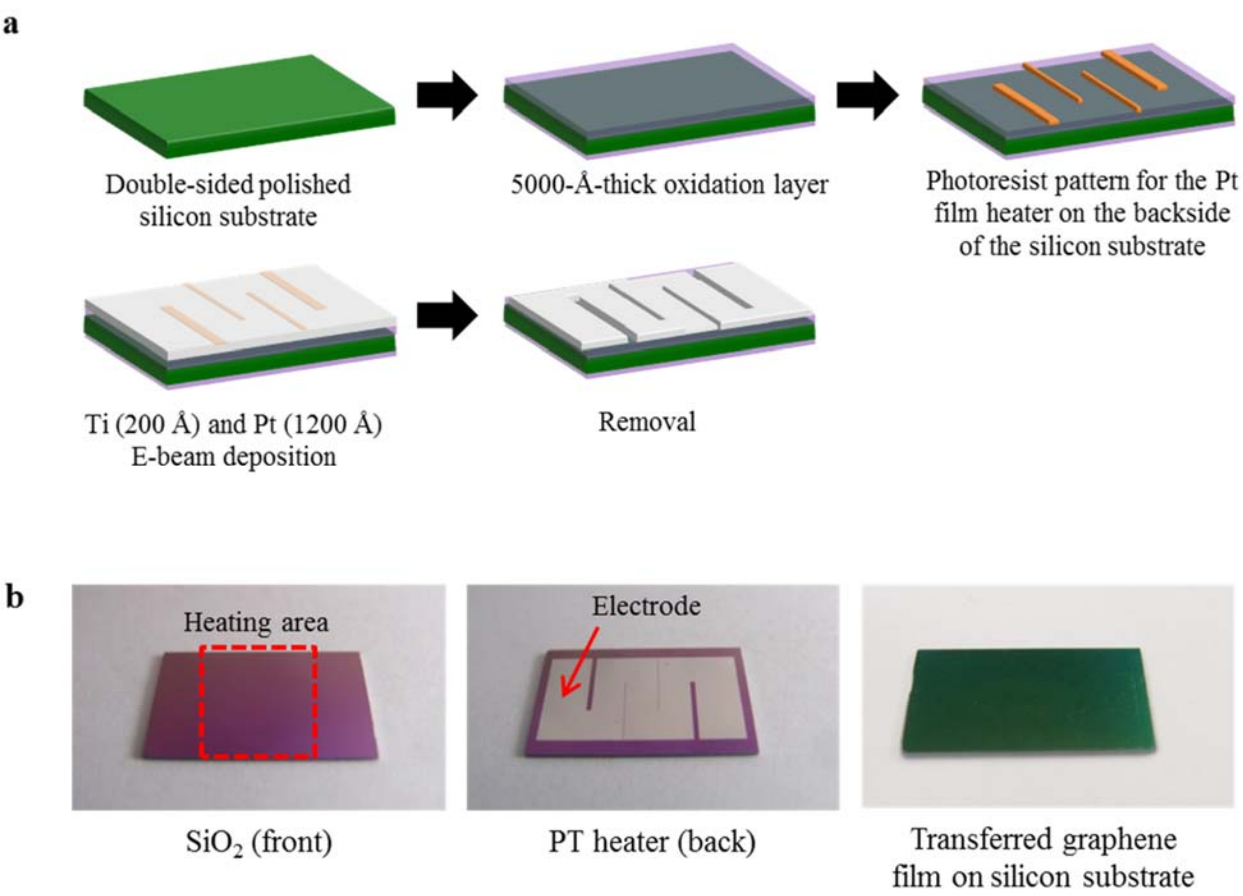

Figure S2 | Fabrication of the silicon heater. a, Schematic images of the fabrication process. **b,** Optical image of the heater.

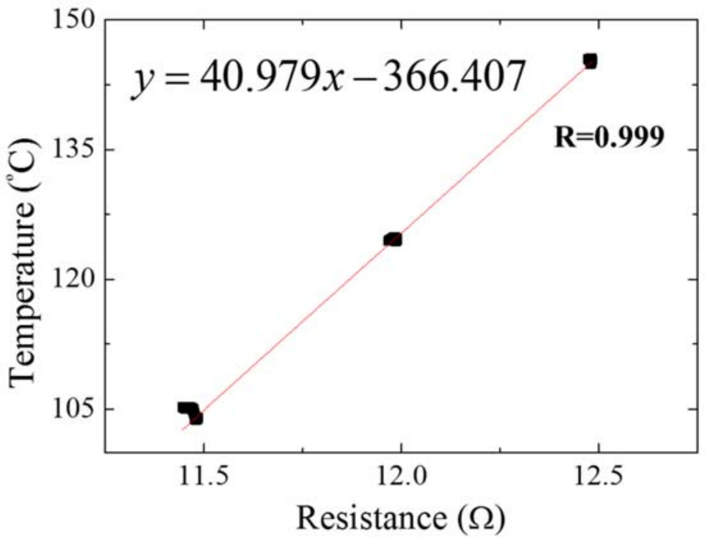

Figure S3 | Heater Calibration. Calibration curve used to calculate the surface temperature.

Prior to the boiling experiments, the working fluid was heated in the pool for 1 hour via a submerged cartridge heater for degassing purposes. During the experiments, the heat flux was increased in 100-kW·m^{-2} steps, with a 2-minute period for steady-state data acquisition. At heat fluxes of less than 100 kW·m^{-2}, a 10-kW·m^{-2} step was used to observe the ONB. When the CHF occurred, the wall temperature increased rapidly, at which time the electrical power was shut down to prevent heater failure.

The analysis of the experimental uncertainty was based on the work of Holman[1]. Using our experimental data, the heat flux can be calculated from

$$q'' = \frac{V_{heater} \cdot V_{ref}}{R_{ref} \cdot A_{heater}} \qquad \text{S-2}$$

The functional form of Equation S-2 is

$$q'' = q''(V_{heater}, V_{ref}, R_{ref}, A_{heater}), \qquad \text{S-3}$$

where A_{heater} is assumed to be constant. The uncertainty analysis becomes

$$U_{q''}^2 = \left(\frac{\partial q''}{\partial V_{heater}}\right)^2 U_{V_{heater}}^2 + \left(\frac{\partial q''}{\partial V_{ref}}\right)^2 U_{V_{ref}}^2 + \left(\frac{\partial q''}{\partial R_{ref}}\right)^2 U_{R_{ref}}^2, \qquad \text{S-4}$$

where $U_{q''}$, $U_{V_{heater}}$, $U_{V_{ref}}$, and $U_{R_{ref}}$ represent the uncertainties in q'', V_{heater}, V_{ref}, and R_{ref}, respectively. Equation S-4 can be simplified to

$$\frac{U_{q''}}{q''} = \sqrt{\frac{U_{V_{heater}}^2}{V_{heater}^2} + \frac{U_{V_{ref}}^2}{V_{ref}^2} + \frac{U_{R_{ref}}^2}{R_{ref}^2}}, \qquad \text{S-5}$$

The general form of the surface temperature is

$$T_{wall} = \frac{1}{a}(R_{heater}) = \frac{1}{a}\left(\frac{V_{heater} \cdot R_{ref}}{V_{ref}}\right). \quad \text{S-6}$$

The uncertainty of the surface temperature can therefore be expressed by

$$\frac{U_{wall}}{\Delta T_{wall}} = \sqrt{\frac{U_{V_{heater}}^2}{V_{heater}^2} + \frac{U_{V_{ref}}^2}{V_{ref}^2} + \frac{U_{R_{ref}}^2}{R_{ref}^2}} \; . \quad \text{S-7}$$

Accounting for the uncertainty in the instruments, the maximum uncertainties in the heat flux and wall temperature were estimated to be less than 1.8% and 3.0%, respectively, over the expected range of the heat flux.

S3. RGO filtration and transfer method

The procedure to synthesize the RGO flakes was as follows. A mixture of 55 mg of GO in 55 mL of DI water was sonicated in a high-intensity ultrasonic processor (Autotune Series, 750 W) for 30 min. The solution was then centrifuged (MF500 centrifuge, Hanil) at 3000 rpm for 30 min. Subsequently, 50 mL of the GO mixture was diluted with 50 mL of distilled water containing 50 μL of hydrazine solution (35 wt.%, Aldrich) and 250 μL of ammonia solution (~30%). After a few minutes of vigorous mixing, the solution was maintained at 95°C for 3 hours.[2] The solubility of RGO is approximately 0.3 mg/mL. The quantity of RGO in the mixture was measured by filtering a known amount of the mixture and weighing the RGO and filter paper. A photograph of the RGO flakes suspended in DI water is shown in Fig. S4a. A TEM image of typical RGO flakes synthesized via the above method is shown in Fig. S4b. Figure S4c shows SAED patterns measured in the area indicated by the box shown in the TEM image. The region was oriented along the [001] axis. The 24 spots in the first ring, corresponding to reflections from the (1100) plane, revealed hexagonal symmetry in the [0001] diffraction pattern.[3] These 24 bright spots represent four highly crystalline sheets of graphene that overlapped with each other. The suspended RGO flakes in the DI water were characterized via AFM measurements, as shown in Fig S4d; the flakes were 0.675 nm thick and 0.5–1.0 μm across. This is consistent with RGO samples prepared via other processes;[2,4] however, it is thicker than a single graphene layer (0.334 nm). Colloidal suspensions of the RGO flakes in DI water were prepared at concentrations of 0.001 wt.%.

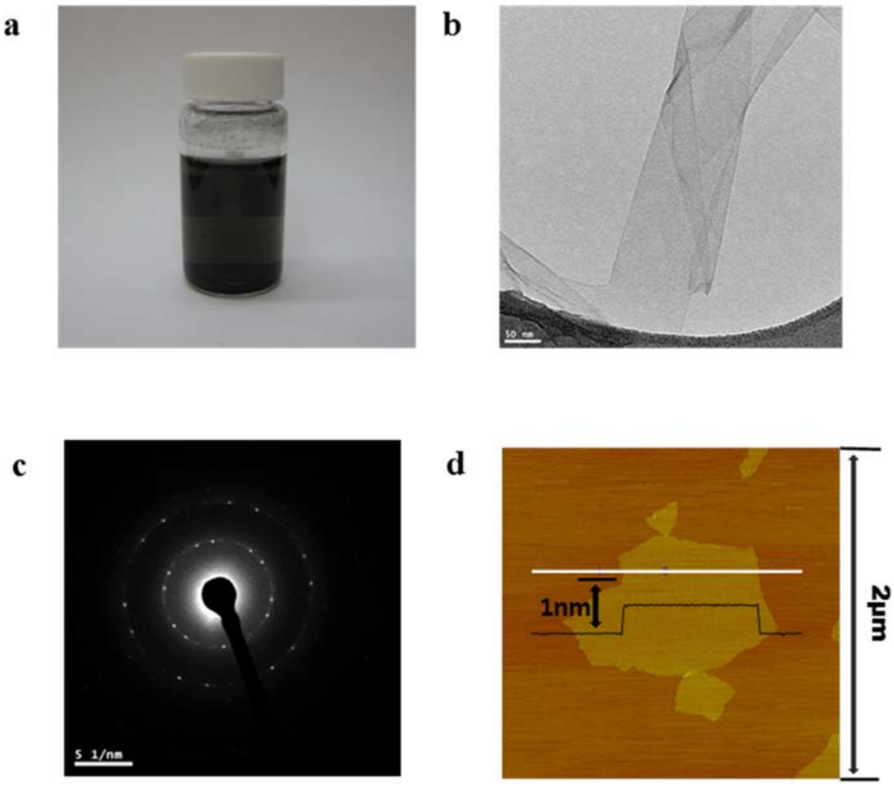

Figure S4 | Characteristics of the RGO flakes. a, RGO colloidal suspension. **b,** TEM images of the folded flakes. **c,** SAED patterns of the two RGO flakes. **d,** AFM image of an RGO flake.

The graphene film was obtained via filtration, which was carried out using a vacuum. As shown in Figure S5, cellulose paper filter with 450-nm-diameter pores was used to filter the RGO flakes from the colloidal suspension. During filtration, the cellulose paper retained the RGO flakes at the surface as the water flowed through the paper, resulting in conformal orientation of the RGO flakes along the filter paper.[5]

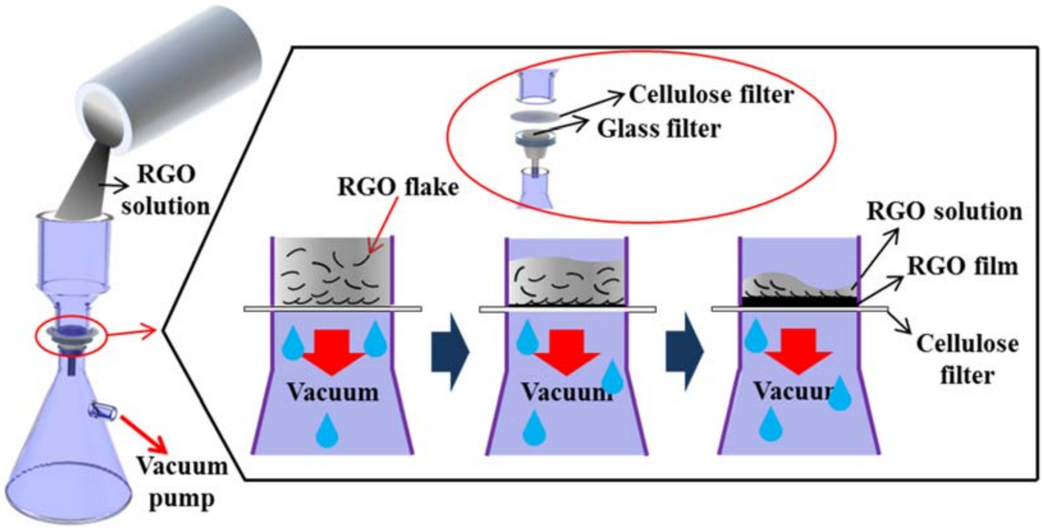

Figure S5 | Formation of RGO via filtration.

Following filtration, the graphene film was on the cellulose filter paper, along with absorbed water. Alumina ceramic blocks were used to press the silicon heater and the cellulose paper together using a vice, as shown in Fig. S6, and placed in an oven at 63°C for 1 hour. The cellulose paper was subsequently peeled off the heater, leaving the graphene film on the heater, as shown in Fig. S2b.

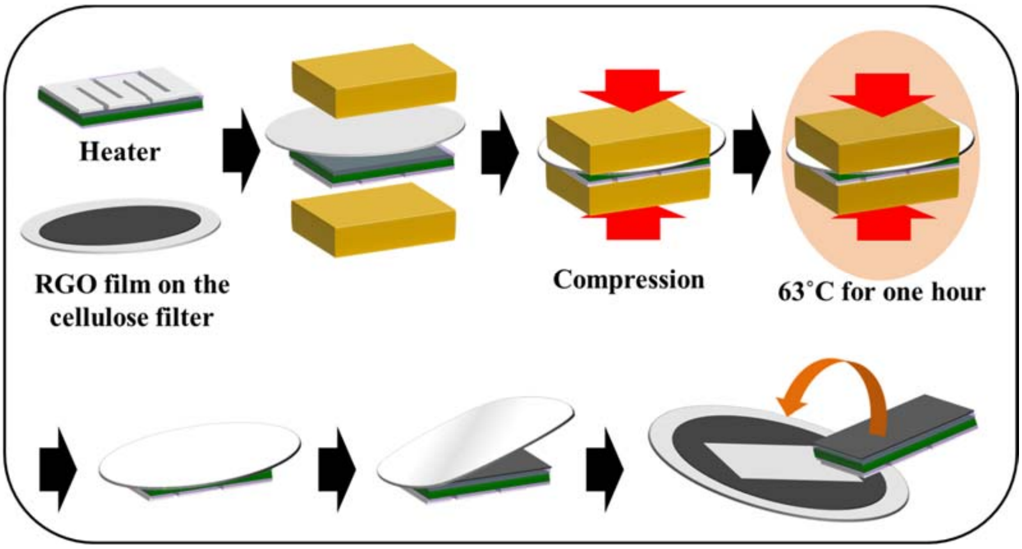

Figure S6 | RGO film transfer method. Schematic images showing the RGO film transfer process.

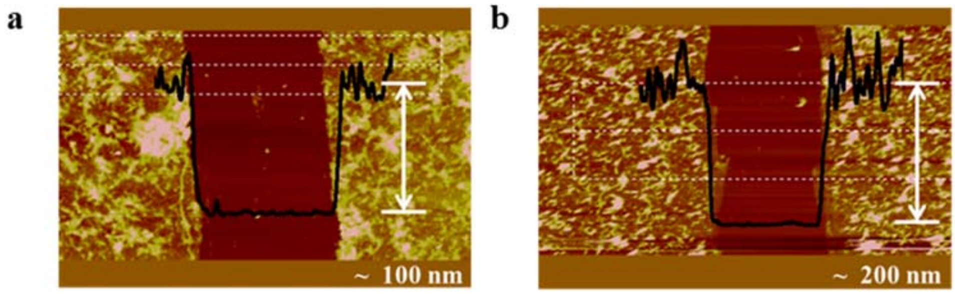

Figure S7 | Thickness measurement of the transferred RGO film. AFM images showing the thickness of the **a,** 100-nm-thick RGO film and **b,** 200-nm-thick RGO film.

The surface of the graphene-coated heater was carefully scratched using a using a syringe needle, and AFM measurements were carried out to measure the thickness of the coated graphene film. Although the thickness of the graphene films could be predicted via a simple calculation based on the quantity of RGO flakes in the colloidal dispersion, it was measured to improve the precision of the data.

S4. CHF experiments and graphene film thickness

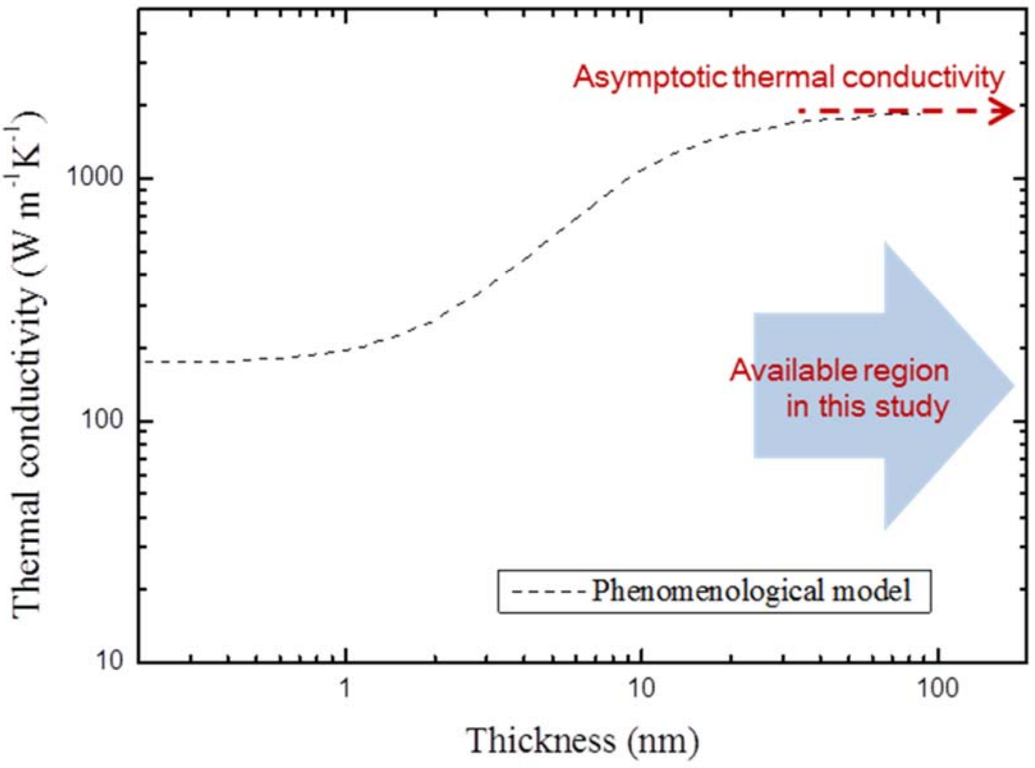

Figure S8 | Thermal conductivity of the graphene film

To calculate the thermal conductivity of the graphene film, we employed the phenomenological model based on the relationship between the thermal conductivity and the thickness of the graphene film. The effective thermal conductivity of the graphene film is given by

$$k = k_0 + (k_{bulk} - k_0)\left[1 - \frac{2\delta}{t}\tanh\left(\frac{t}{2\delta}\right)\right], \qquad \text{S.9}$$

where k_0 is an approximately constant value with zero thickness, k_{bulk} is the thermal conductivity of bulk graphite, δ is the characteristic length, and t is the total thickness of the film. This phenomenological model was fitted to the data shown in Fig. S8.

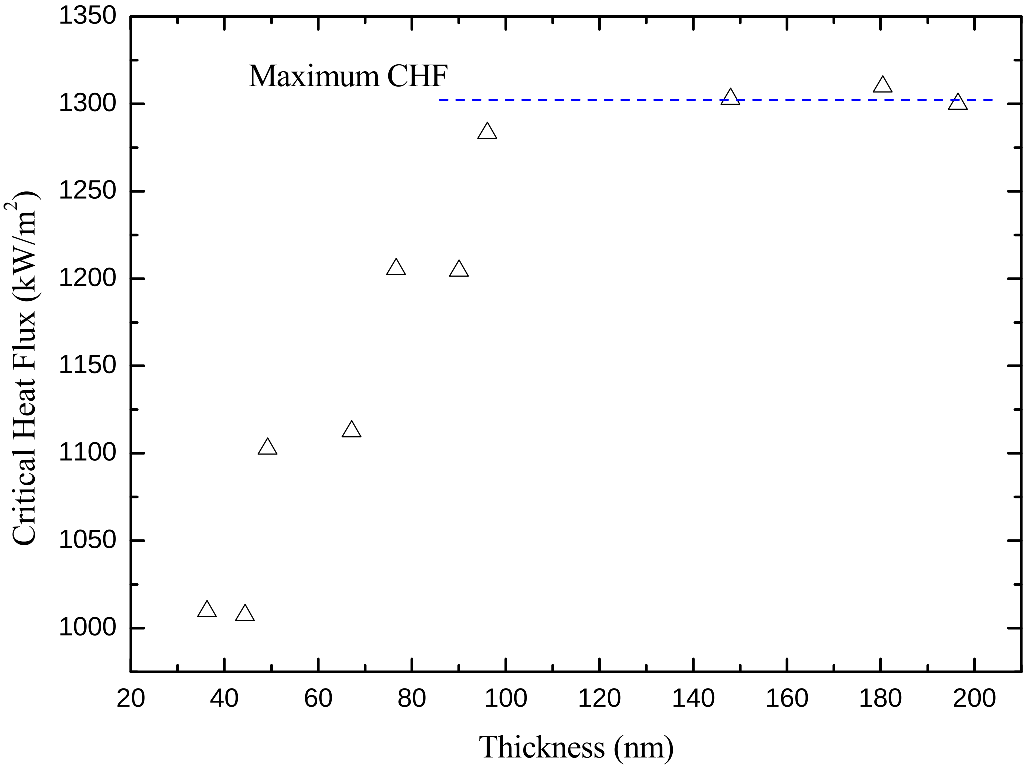

Figure S9 | Critical heat flux as a function of the thickness of the graphene film.

S5. Preparation of IR high-speed visualization

Figure S10a shows schematic diagrams of the fabrication process for the silicon heater used for the IR visualization. Figure S10b shows the fabricated heater. This heater was designed for high-speed IR thermometry, which was used to acquire the temperature distribution of the heater surface during nucleate boiling. The key feature of the IR-visualization technique was the use of a heater material that was transparent to IR radiation (i.e., optical grade silicon wafer), and an IR emission layer that was opaque to IR radiation (i.e., the ITO layer). An optical-grade double-sided polished silicon wafer was used as the base substrate of the IR-visualization heater. On the front side, a thermally grown 2-μm-thick SiO_2 layer was prepared to electrically insulate the ITO layer from the silicon substrate. The 700-nm-thick ITO layer was then sputtered onto the heater, and the front surface was finished by depositing another 1-μm SiO_2 layer via plasma-enhanced chemical vapor deposition (PE-CVD). The back of the heater had electrode pads for electrical connections to the DC power supply. In order to prepare the electrode pads, the 2-μm SiO_2 layer on the back of the test heater was removed by immersing into a 6:1 mix of buffered oxide etchant (BOE), and 6-nm-thick Ti and 100-nm-thick Au layers were deposited using E-beam evaporation to form contacts. The heater had an effective boiling area of 20 mm × 10 mm in the center.

Figure S10c shows a schematic diagram of the IR visualization during boiling. A pool chamber similar to that shown in Fig. S1a was used for the IR visualization experiment during saturated boiling. The test sample was placed on the bottom of the pool chamber. A high-speed IR camera (FLIR SC6000) was placed below the heater, and the working fluid was located on top of the heater. The IR camera was used to image IR radiation emitted from the ITO layer. When the temperature of a local area on the heater changed, the IR camera detected this variation in the IR intensity. The spatial resolution of the camera was approximately 65 μm, and the time resolution was 1 ms.

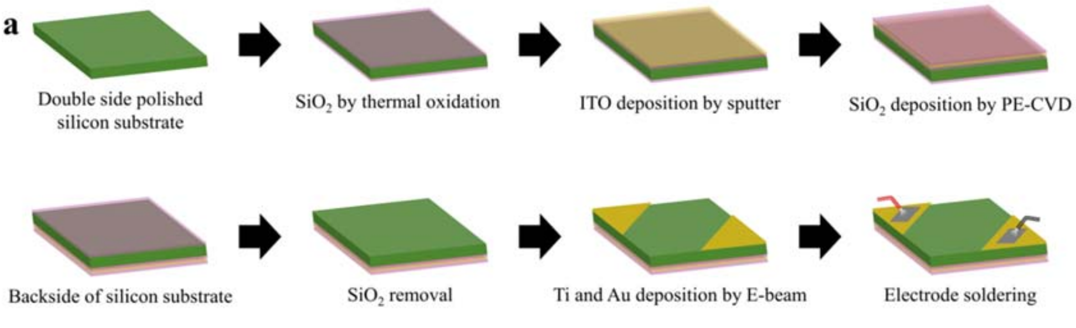

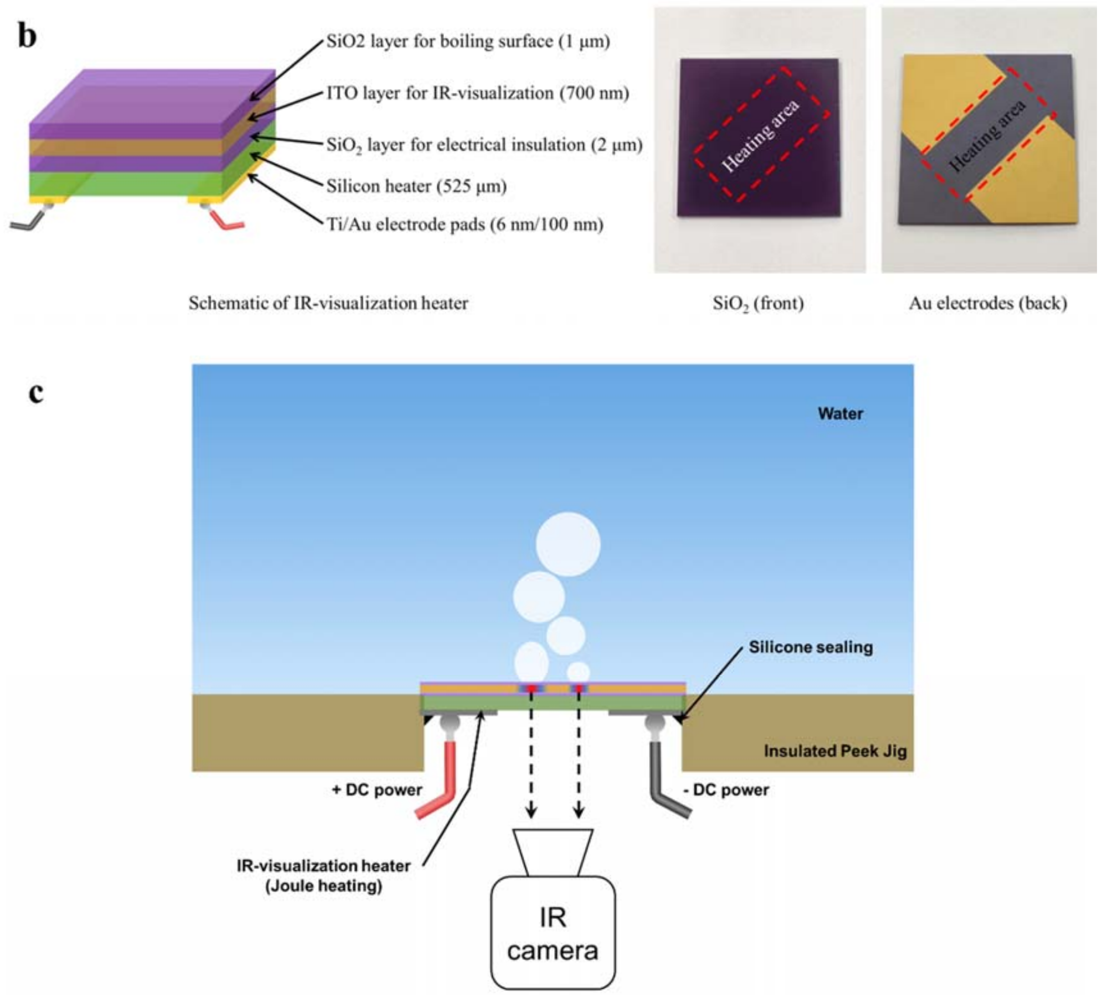

Figure S10 | Silicon heater with an ITO layer and IR visualization. a, Schematic diagrams showing the fabrication process. **b,** A schematic diagram of the heater used in the IR visualization experiments. **c,** Schematic diagram of the setup used for the for the IR visualization.

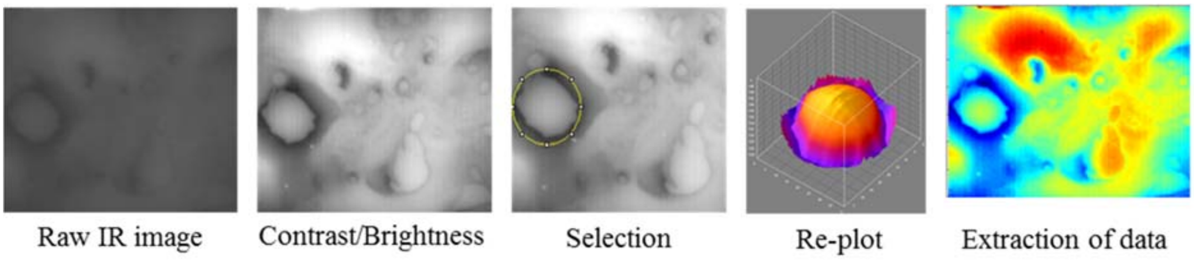

Figure S11 | Extraction of temperature data from the IR images

The temperature distribution was extracted from the IR images as a matrix of temperatures at each pixel using Image-J, which is a software for image analysis. Initially, dry areas were identified in order to determine the effects of the graphene film during boiling, as shown in Fig. S11. To compare the bare silicon surface and the 50-nm-thick graphene-coated surface, temperature distributions were extracted at a heat flux of 98% of the CHF of the bare silicon heater, under which conditions dry areas appeared on the bare silicon surface. To compare the 15-nm-thick graphene-coated surface with the 50-nm-thick graphene-coated surface, temperature distributions were extracted at 80% and 100% of the CHF of the 15-nm-thick graphene-coated surface. IR images are shown with the time relative to the dry area being detected. The centers of the bubble nucleation sites were selected to observe the time variation of the temperature because the invervention of the water were less significant at the center. Sufficient nucleation sites were selected at 40% of the CHF of the bare silicon heater to avoid overheating. At this heat flux, changes in temperature were analyzed as a function of time, and the rate of the change of temperature was calculated from nucleation to extinction. Nucleation sites with similar sizes were selected in order to compare the temperature gradients on the different surfaces at 98% of the CHF of the bare silicon heater. At these sites, the temperature was extracted along the centerline of the bubble, and the distances were normalized for the sake of comparison. The temperature gradients were calculated using these 1-D data arrays.

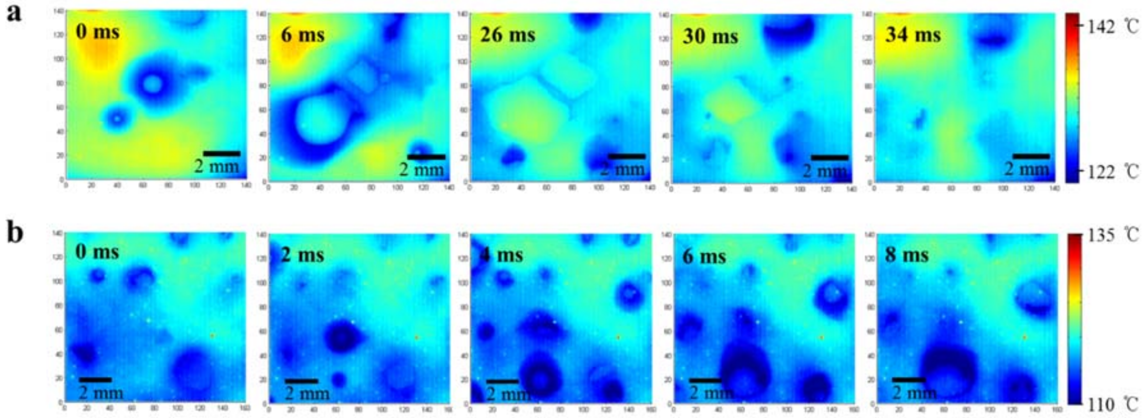

Figure S12. IR visualization during boiling at 40% of the CHF of the bare silicon heater. **a,** Bare silicon and **b,** a 50-nm-thick graphene film.

Figure S12 shows the temperature distribution on the bare silicon and the graphene-coated heated surfaces at the same heat flux. The density of nucleation sites was larger on the graphene-coated heater, and the bubble departure diameter was smaller, with a higher frequency of bubble generation than on the bare silicon heater. The heat flux due to boiling heat transfer can be described using a micro-convection heat transfer model,[6] i.e.,

$$\therefore\ h \sim \frac{1}{A}\sum_{N_a} f^{1/2} \cdot D_d^2 \ , \qquad \text{S.10}$$

where f is the frequency of bubble departure, D_d is the diameter of the bubble at departure, N_a is the density of the nucleation sites, A is the area of the heated surface, and the summation is over the number of nucleation sites N_a. The bubble diameter was estimated from the baseline diameter of the bubble from the IR visualization data.

The bare surface exhibited slightly larger bubbles on departure than the graphene coated-surface. At 40% of the CHF of the bare silicon heater, the bubble diameter was in the range 1.49 mm $< D_d <$ 4.55 mm on the bare surface, and 0.25 mm $< D_d <$ 2.71 mm on the 50-nm-thick graphene-coated heater surface. The 50-nm-thick graphene-coated surface exhibited a three-fold increase in the density of nucleation sites compared with the bare silicon surface. The frequency of bubble generation was in the range 27.03 $s^{-1} < f <$ 200 s^{-1} on 50-nm-thick graphene-coated surface, and 14.29 $s^{-1} < f <$ 45.45 s^{-1} on the bare silicon surface, as shown in Fig. S12. Consequently, the heat flux due to boiling heat transfer was 1.8 times larger on the graphene-coated surface than on the bare silicon surface.

S6. Bubble dynamics from the IR high-speed visualization

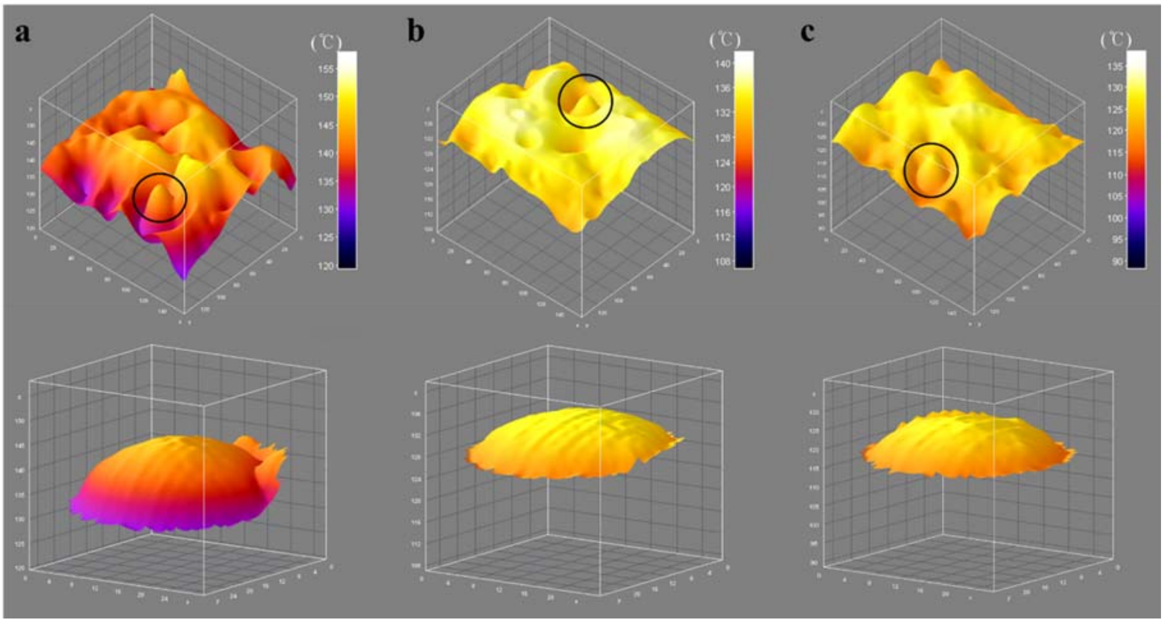

Figure S13 | Temperature distribution of selected bubble sites. a, Bare silicon surface. **b,** 15-nm-thick graphene-coated surface. **c,** 50-nm-thick graphene-coated surface. All at 98% of the CHF of the bare silicon surface.

Figure S13 shows the temperature distribution at bubble nucleation sites for each surface at 98% of the CHF of the bare silicon surface. The black circles in the large-area plots indicate the nucleation sites shown. These nucleation sites were similar in size; however, they exhibited significantly different temperature gradients.

Location	Temperature (°C)		dT / dx		dT / dt	$\nabla^2 T$
Time (s)	0	0.001	0	0.001	0 – 0.001	0 – 0.001
–0.0004669	116.8179	124.384				
–0.0004002	119.2173	125.2877	35973.01	13548.73	6070.4	
–0.0003335	120.8762	125.3345	24871.06	701.6492	4458.3	−166446012.4
–0.0002668	121.9618	125.7686	16275.86	6508.246	3806.8	−128863604.2
–0.0002001	122.6728	126.0973	10659.67	4928.036	3424.5	−84200778.17
–0.0001334	123.6298	126.9332	14347.83	12532.23	3303.4	55294691.48
–0.0000667	123.5837	126.7279	–691.154	–3077.96	3144.2	−225471971.7
0.0000000	123.4735	126.8112	–1652.17	1248.876	3337.7	−14408088.31
0.0000667	123.1532	126.6756	4802.099	2032.984	3522.4	96765710.1
0.0001334	122.8186	126.8076	5016.492	–1979.01	3989	3214284.912
0.0002001	121.915	126.3034	13547.23	7559.22	4388.4	127897071
0.0002668	120.4445	125.9248	22046.48	5676.162	5480.3	127425043.1
0.0003335	118.0653	125.6139	35670.16	4661.169	7548.6	204253195.7
0.0004002	117.1019	125.472	14443.78	2127.436	8370.1	−318236683.8

Table S1. Wall temperatures under a bubble, used to calculate the thermal diffusivity of the bare silicon heater.

Location	Temperature (°C)		dT / dx		dT / dt	$\nabla^2 T$
Time (s)	0	0.002	0	0.002	0 – 0.002	0 – 0.002
−0.0008905	132.3712	143.1213				
−0.0008220	135.0734	143.9824	39448.18	12570.8	4454.5	
−0.0007535	137.591	144.1912	36753.28	3048.175	3300.1	−39341467.31
−0.0006850	139.3022	144.9883	24981.02	11636.5	2843.05	−171857850.7
−0.0006165	140.3055	145.1226	14646.72	1960.584	2408.55	−150865789.3
−0.0005480	141.0722	145.2807	11192.7	2308.029	2104.25	−50423570.78
−0.0004795	141.8493	145.6799	11344.53	5827.737	1915.3	2216420.694
−0.0004110	142.6526	146.2147	11727.01	7807.299	1781.05	5583675.209
−0.0003425	143.1888	146.4493	7827.737	3424.818	1630.25	−56923650.7
−0.0002740	143.7434	146.6414	8096.35	2804.38	1449	3921359.689
−0.0002055	144.1643	146.6707	6144.526	427.7372	1253.2	−28493792.96
−0.0001370	144.8564	147.2261	10103.65	8108.029	1184.85	57797431.94
−0.0000685	145.1027	147.144	3595.62	−1198.54	1020.65	−95007725.5
0.0000000	145.3104	147.1683	3032.117	354.7445	928.95	−8226330.652
0.0000685	145.2845	147.0157	378.1022	2227.737	865.6	−38744738.66
0.0001370	145.4594	147.0842	−2553.28	−1000	812.4	−42793968.78
0.0002055	145.3802	146.9109	1156.204	2529.927	765.35	54153124.83
0.0002740	145.2091	147.0189	2497.81	−1576.64	904.9	19585486.71
0.0003425	144.8606	146.8199	5087.591	2905.109	979.65	37807022.22
0.0004110	144.6631	146.8141	2883.212	84.67153	1075.5	−32180723.53
0.0004795	144.2085	146.6054	6636.496	3046.715	1198.45	54792476.96

0.0005480	143.6912	146.2845	7551.825	4684.672	1296.65	13362459.37
0.0006165	143.0982	146.0573	8656.934	3316.788	1479.55	16132985.24
0.0006850	142.493	145.8639	8835.036	2823.358	1685.45	2600031.968
0.0007535	141.7559	145.591	10760.58	3983.942	1917.55	28110181.68
0.0008220	141.0488	145.3825	10322.63	3043.796	2166.85	−6393521.232
0.0008905	140.075	145.0461	14216.06	4910.949	2485.55	56838403.75

Table S2. Wall temperatures under a bubble, used to calculate the thermal diffusivity of the graphene-coated heater.

References

1. Holman JP. *Experimental Methods for Engineers*, 7th edn. McGraw-Hill, 2001.

2. Hummers Jr WS, Offeman RE. Preparation of graphitic oxide. *J. Am. Chem. Soc.* **80,** 1339-1339 (1958).

3. Li D, Müller MB, Gilje S, Kaner RB, Wallace GG. Processable aqueous dispersions of graphene nanosheets. *Nat. nanotechnol.* **3,** 101-105 (2008).

4. Ahn HS, Jang J-W, Seol M, Kim JM, Yun D-J, Park C*, et al.* Self-assembled foam-like graphene networks formed through nucleate boiling. *Sci. Rep.* **3** (2013).

5. Liang Q, Yao X, Wang W, Liu Y, Wong CP. A three-dimensional vertically aligned functionalized multilayer graphene architecture: an approach for graphene-based thermal interfacial materials. *ACS nano* **5,** 2392-2401 (2011).

6. Mikic B, Rohsenow W. A new correlation of pool-boiling data including the effect of heating surface characteristics. *J. Heat Transf.* **91,** 245-250 (1969).

7. Jang W, Chen Z, Bao W, Lau CN, Dames C. Thickness-dependent thermal conductivity of encased graphene and ultrathin graphite. *Nano Lett.* **10,** 3909-3913 (2010).
